# Using pyrene to probe the effects of poloxamer stabilisers on internal lipid microenvironments in solid lipid nanoparticles†

**DOI:** 10.1039/d0na00582g

**Published:** 2020-10-19

**Authors:** Jessica M. Taylor, Kyle Scale, Sarah Arrowsmith, Andy Sharp, Sean Flynn, Steve Rannard, Tom O. McDonald

**Affiliations:** Department of Chemistry, University of Liverpool Crown Street Liverpool L69 7ZD UK Thomas.Mcdonald@liverpool.ac.uk; Department of Cellular and Molecular Physiology, Institute of Translational Medicine, Liverpool Women's Hospital, University of Liverpool Crown Street Liverpool L8 7SS UK; Department of Women's and Children's Health, Liverpool Women's Hospital, University of Liverpool Crown Street Liverpool L8 7SS UK

## Abstract

Solid lipid nanoparticles (SLNs) have proved to be effective nanocarriers with many advantages over other non-lipid-based systems. The development of new SLN formulations is often hindered through poor drug loading capacity and time-consuming optimisation of lipid/stabiliser combinations. One challenge in the development of new SLN formulations is understanding the complex interactions between amphiphilic stabilisers and hydrophobic lipids; the nature of these interactions can significantly impact SLN properties, including the internal polarity within the nanoparticle core. Herein, we report the use of pyrene to probe the internal lipid microenvironment inside SLNs. We investigate the effect of using different poloxamer stabilisers on the internal polarity of SLNs formed using the common solid lipid, Compritol 888 ATO. We show that the polarity of the internal lipid environment is modified by the length of the poly(propylene oxide) (PPO) block of the poloxamer stabiliser, with longer PPO blocks producing SLNs with less polar lipid cores. Blending of stabilisers could also be used to tune the polarity of the core lipid environment, which may allow for adjusting the polarity of the lipid to assist the loading of different therapeutics.

## Abstract 2

Pyrene can be used to investigate the internal polarity inside solid lipid nanoparticles; small changes in the structure of the poloxamer surfactants can influence the polarity inside the core.

## Graphical abstract

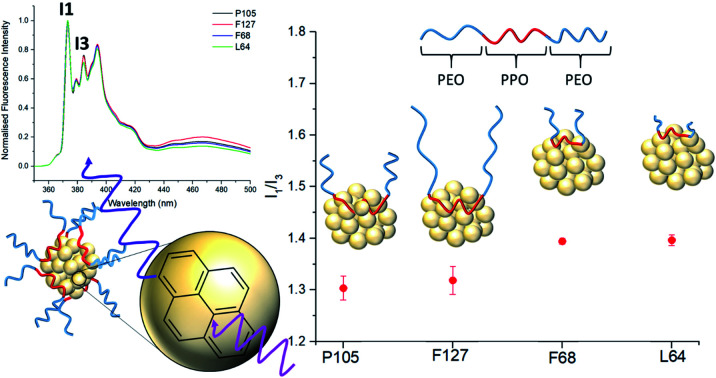

Nanomedicine has offered the potential to improve the pharmacological profiles of poorly performing or low aqueous solubility medicines.^1,2^ Solid lipid nanoparticles (SLNs) are a type of nanomedicine that is composed of a lipid core that remains solid at body temperature and an active pharmaceutical ingredient (API).^3–5^ The hydrophobic lipid/API cores are stabilised by amphiphilic stabilisers such as poloxamers (often known by the brand names Pluronic®, Poloxamer® or Synperonic®), which are triblock copolymers of polyethylene oxide-polypropylene oxide-polyethylene oxide (PEO-PPO-PEO) (Fig. 1).^6,7^ They have shown a high degree of versatility as stabilisers due to their low biological toxicity and ability to enhance the solubilisation of lipophilic compounds.^8^ Poloxamers have been shown to improve the pharmacological profiles of several poorly performing small molecule drugs and are the most common stabilisers used in SLN formulations.^6,7^ Compritol 888 ATO is one of the most commonly used lipids within SLNs.^9^ This solid lipid is a mixture of mono, di and triglycerides of behenic acid (Fig. 1), and has been well documented showing high encapsulation efficiencies (EE) of several hydrophobic entities. EE is a parameter relating to the amount of drug successfully entrapped within the nanocarrier, thus within the lipid core of SLNs. Examples of reported Compritol 888 ATO SLN systems with significant EE values have included the encapsulation of triamcinolone acetonide (EE = 99%), acyclovir (EE = 56–81%) and indomethacin (EE = 72%).^10–12^ Interestingly, in addition to the use of Compritol 888 ATO in the mentioned formulations, the authors have also explored a mutual secondary excipient, a poloxamer, Pluronic® F68 as a potential amphiphilic stabiliser. It is known that the incorporation of APIs within the core of SLNs disrupts the natural crystallinity of the lipid, potentially facilitating a more controlled, site specific release of the API.^13–16^ SLNs can be produced by a number of methods including hot, cold or high speed homogenisation,^17^ ultrasonication, spray drying and nanoprecipitation.^18,19^ Of these methods, nanoprecipitation is a particularly attractive method due to practical simplicity and scalability, as well as time and cost effectiveness.^20–24^ Nevertheless, there are still some challenges to address in the development of SLNs. A common drawback is as a result of low drug loading capacity compared to some other nanocarrier systems.^13,25–27^ Drug loading, a different parameter to encapsulation efficiency, is typically given as mass percentage of the drug with respect to the total combined mass of the lipid, drug and stabiliser; the lower the drug loading of a formulation the greater the amount of carrier materials that will be administered along with the drug dose.^28^ This limitation is classically associated with poor compatibility between the crystalline lipid and the API mixture resulting in phase separation in the nanoparticle core.^13,29^ To address these drawbacks, considerable optimisation of the SLN formulation is required. Typically, this involves time-consuming trial and error based methods for the exploration of different excipients and a screening process to identify their optimum compositions; particularly to reduce internal phase separation and increase drug loading. It is known that stabilisers can influence the internal polarity of the environment inside nanoparticles, therefore with potential to modify the drug loading behaviour through a change in the lipid core microenvironment.^30^ Thus, there is a need to understand how the selection of stabilisers can influence the core polarity of a solid lipid, as this understanding could potentially be used to tune the lipid core polarity to enhance the lipid/API affinity and reduce API partitioning. For example, if the relevant API is highly hydrophobic, the lipid environment should be hydrophobic also; however, if the API contains some polar character, then the lipid environment might be tuned to accommodate a greater polarity in the lipid core microenvironment. Changes in the polarity of the lipid core can be observed through implementing pyrene as a fluorescent probe. Pyrene is a small molecule that has a unique, polarity dependent fluorescence emission, which consists of five defined vibrational emission bands (*I*_1_–*I*_5_). The ratio of the first (*I*_1_) and third (*I*_3_) vibronic bands fluctuates in response to changes in pyrene's external environment, with lower *I*_1_/*I*_3_ ratios obtained in low-polarity environments.^6,31–36^ As pyrene is highly hydrophobic, it partitions into the lipid phase in SLN systems and therefore provides relevant information on the polarity of the lipid core and the effect that different stabilisers have on this environment.

**Fig. 1:**
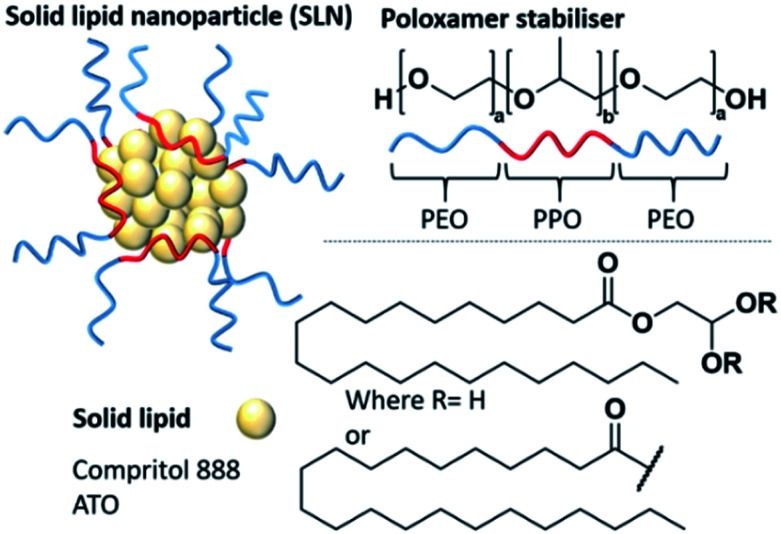
Solid lipid nanoparticles (SLNs) composed on an amphiphilic stabiliser (in this case a poloxamer) and a solid lipid core. Compritol 888 ATO is a common solid lipid and is made of a mixture of mono, di and triglycerides of behenic acid.

In this study, we have chosen to investigate the influence of poloxamer stabilisers and their effect on the polarity of the core made of the common solid lipid, Compritol 888 ATO (Fig. 1). Within the family of poloxamer stabilisers (>50 polymers) there is huge variability in the hydrophilic–lipophilic balance (HLB) values, molecular weight and PPO/PEO ratios. We have therefore selected four poloxamers, commonly used in SLN formulations (Pluronic® P105, F127, L64 and F68), to compare the influence of HLB, molecular weight and PPO/PEO ratio (Table 1) on their ability to produce stable SLNs and influence the polarity of the SLN core microenvironment. Firstly, SLNs consisting of 100% solid lipid core and no pyrene, which we will term ‘blank’ SLNs, were prepared in triplicate to show reproducibility and allow comparison of all physical properties of the SLNs with and without pyrene. The following nanoprecipitation method was used; Compritol 888 ATO (18 mg, 4.5 w/v%) was heated to 82 °C in 1-propanol (4 mL) for 5 minutes. Compritol 888 ATO is a high melting point solid lipid with a cited melting point of ∼70 °C.^37^ The hydrophobic phase was heated ∼12 °C higher than the average melting point to ensure complete melting and homogeneity within the hydrophobic phase for injection. The aqueous phase (20 mL) containing the poloxamer stabiliser (16 mg, 0.8 w/v%) was agitated using mechanical stirring at 350 rpm and warmed to 26 °C. The heated hydrophobic phase was then rapidly injected into the aqueous phase and stirred for a further 5 minutes. To obtain pyrene loaded particles, the method was repeated but pyrene (0.034 mg) was incorporated into the hydrophobic lipid phase in 1-propanol prior to injection. These methods resulted in either blank SLNs with a mass composition of Compritol 888 ATO (53 wt%) and poloxamer stabilisers (47 wt%). Such compositions of lipid to stabiliser are typical in the literature, with values in the range of 50–77 wt% stabiliser reported.^38–41^ When pyrene-SLNs were prepared, an additional 0.034 mg of pyrene was present at 0.1 wt% of the total solid mass. All nanoprecipitations resulted in a dispersion in a water/1-propanol mixed solvent system (5 : 1). SLNs were analysed by dynamic light scattering (DLS) in order to determine the size distribution and mean intensity-derived average hydrodynamic diameter (*D*_z_). Fluorescence emission spectroscopy was undertaken to assess the internal polarity within the core of SLNs. For full experimental details see the ESI, Section E1.†

**Table 1:** The poloxamers stabilisers chosen to investigate their effect on lipid core polarity. This table contains cited HLB and CMC data from Figueiras *et al.*^42^ Poloxamers are ordered in increasing *I*_1_/*I*_3_ values with respect to SLN dispersions

Poloxamer (Pluronic®)	Formula	Average *M*_w_ (g mol^−1^)	CMC at 25 °C (M)	HLB value	PPO/PEO ratio	*I*_1_/*I*_3_ Pluronic®-micellar solutions	*I*_1_/*I*_3_ SLN dispersions	*e*/*m* ratio of SLN dispersions
P105	PEO_37_-PPO_56_-PEO_37_	6500	6.2 × 10^−6^	15	0.76	1.63 ± 0.07	1.30 ± 0.02	0.18
F127	PEO_100_-PPO_65_- PEO_100_	12 600	2.8 × 10^−6^	22	0.33	1.66 ± 0.01	1.32 ± 0.03	0.21
F68	PEO_76_-PPO_29_-PEO_76_	8400	4.8 × 10^−4^	29	0.20	1.72 ± 0.02	1.39 ± 0.005	0.17
L64	PEO_13_-PPO_30_-PEO_13_	2900	4.8 × 10^−4^	15	1.20	1.70 ± 0.001	1.40 ± 0.01	0.15

Each poloxamer was investigated for its effect on *D*_z_, polydispersity index (PDI) and changes in internal core polarity. Particle size analysis showed monomodal particle size distributions for both blank and pyrene-loaded SLNs. All SLNs had very similar *D*_z_ values at 230 ± 27 nm for unloaded SLNs, and 242 ± 48 nm for pyrene-loaded SLNs (this difference in *D*_z_ was within the sample-to-sample variability), regardless of the poloxamer used as the stabiliser (Fig. 2 and ESI, Fig. S1†). This therefore suggests that the different poloxamer stabilisers had little influence on formation of the SLNs and that there was no significant difference in particle size upon the incorporation of pyrene. This meant that any differences in the internal polarity of the SLNs prepared with the different stabilisers can be correlated to the environment inside the lipid core rather than differences in the size of the nanoparticles.

**Fig. 2:**
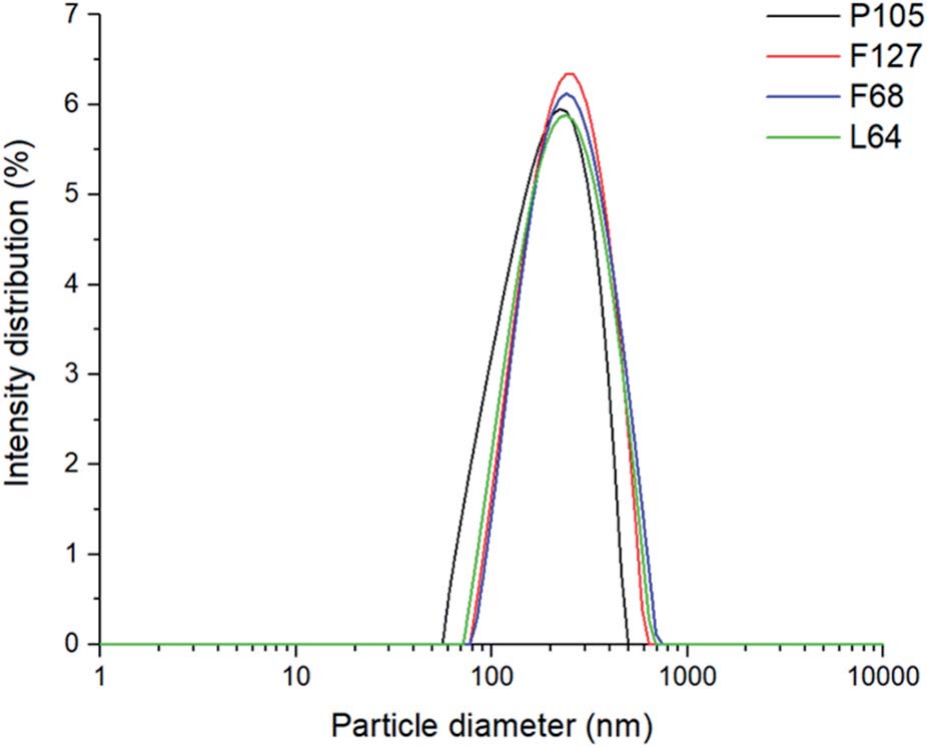
The monomodal size intensity distributions of pyrene loaded SLNs.

The fluorescence emission behaviour of pyrene and its link to polarity was investigated with the comparison of three different solvent environments: pure water, water and 1-propanol mixed (5 : 1 v/v) (the environment formed after nanoprecipitation) and pure 1-propanol. (ESI, Fig. S2†). The resulting *I*_1_/*I*_3_ values showed the differences in the polarities provided by the different solvents with values of 2.18 ± 0.09, 1.78 ± 0.003 and 1.21 ± 0.002 for pure water, water and 1-propanol mixed (5 : 1) and pure propanol respectively. Although *I*_1_/*I*_3_ values of pyrene in different organic systems vary throughout the literature, the experimental values found in this study are similar to those by Dong *et al.* indicating respective *I*_1_/*I*_3_ values of 1.87 and 1.09 for water and 1-propanol.^43^ All subsequent samples were measured with a mixed solvent continuous phase of water and 1-propanol mixed (5 : 1) to study the SLNs immediately after synthesis; variation of the *I*_1_/*I*_3_ ratio from a value of 1.78 may therefore be correlated to the impact of the different poloxamers. Firstly, nanoprecipitations of pyrene were carried out into the poloxamer solutions without lipid present to form micellar solutions. All the poloxamers were used at the same concentration (0.8 mg mL^−1^) as used for SLN preparation. The resulting micellar solutions were significantly less turbid than SLN dispersions (ESI, Fig. S3†). Comparison of the *I*_1_/*I*_3_ values for pyrene showed that the micellar environments provided very similar internal polarities, as may be expected given that they possess chemically identical PPO micelle cores (*I*_1_/*I*_3_ = 1.63–1.72; ESI, Fig. S4†). Out of the four different poloxamers F68 and L64, the two stabilisers with the shortest PPO blocks (Fig. 3A), showed the most polar internal environments (Table 1). Pyrene-loaded SLNs were then prepared using the four different poloxamer stabilisers and their *I*_1_/*I*_3_ values were recorded. The incorporation of the lipid resulted in a considerable reduction in the *I*_1_/*I*_3_ values to 1.30–1.40 for all poloxamer stabilisers compared to the micellar solutions (Fig. 3B). This significant difference in the *I*_1_/*I*_3_ values could potentially be a result of the average fluorescence signal from the pyrene encapsulated in the lipid cores of the SLNs and any pyrene encapsulated in micelles that might be present in the sample (although none were detected in the DLS measurements). In order to investigate this the samples were centrifuged and the supernatant was analysed in the absence of the SLNs (ESI, Fig. S5†). Any fluorescence from the supernatant would represent the pyrene contained in the micelles. However, the supernatant was found to display very limited fluorescence with an ill-defined emission spectra. This finding supports that the pyrene was predominantly encapsulated within the lipid cores of the SLNs, independent of the stabiliser used.

**Fig. 3:**
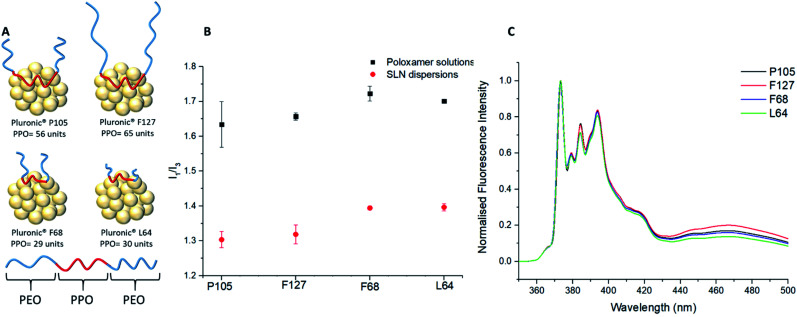
The fluorescence emission behaviour of SLNs with different poloxamer stabilisers. (A) Schematic representation on the different poloxamers and their interaction with a lipid core. (B) Comparison of *I*_1_/*I*_3_ data between different poloxamer stabilisers in the presence of a lipid core or as aqueous solution forming micelles. (C) Fluorescence emission spectra for four different samples of SLNs prepared with for each poloxamer stabiliser (normalised against *I*_1_). Shows the difference in *I*_1_/*I*_3_ values for each poloxamer stabiliser, emphasising the importance of the lipid core from key differences in the excimer emission (440–500 nm).

The substantial difference in the pyrene fluorescence between aqueous micellar solutions and SLNs can therefore be attributed to the non-polar nature of Compritol 888 ATO and its ability to entrap hydrophobic molecules between the C22 alkyl chains.^9,37,44^ As with the *I*_1_/*I*_3_ data for the micelles, it was clear that there were noticeable similarities in the *I*_1_/*I*_3_ values for F68 and L64 compared to F127 and P105 SLN dispersions. In all cases, it would be expected that the PPO blocks of the poloxamers would adsorb and interact with the lipid core, while the PEO chains will extend into the continuous phase and provide steric stabilisation. It was apparent that the molecular weight of the hydrophobic PPO blocks is a dominant factor for controlling the polarity inside the lipid cores of the SLNs. The poloxamers with longer hydrophobic blocks, F127 (PPO = 65) and P105 (PPO = 56) showed significantly lower *I*_1_/*I*_3_ ratios in comparison to poloxamers with shorter PPO blocks, L64 (PPO = 30) and F68 (PPO = 29). This change in polarity can be attributed PPO chains not only adsorbing to the surface of the SLN but also partitioning into the lipid core and reducing the internal polarity. While F127 possesses a longer PPO block compared to P105, it did not have the lowest average *I*_1_/*I*_3_ value at 1.32 ± 0.03 *vs.* P105 at 1.30 ± 0.02. This can potentially be attributed to the much larger PEO block of F127 (PEO = 200) compared to P105 (PEO = 74) causing increased steric hindrance between the F127 units which might potentially limit the extent to which PPO block of F127 can associate with the lipid core.

The hydrophilic–lipophilic balance (HLB) is often used to predict the properties and potential uses of stabilisers.^45^ As shown in Table 1, Pluronic® P105 and L64 have the same cited HLB value on the Griffin scale of 15.^42^ However, P105 and L64 stabilised SLNs had significantly different *I*_1_/*I*_3_ values of 1.30 ± 0.02 and 1.40 ± 0.01 and therefore it can be concluded the HLB is not a viable parameter to predict microenvironment polarity.

The difference in lipid/stabiliser behaviour of these SLN samples was also apparent from the excimer emission which was observed at 440–500 nm in the pyrene emission spectra (Fig. 3C). The excimer emission is a fundamental feature in pyrene fluorescence which suggests that the individual pyrene molecules have a spatial proximity ≤3.5 Å.^46^ The poloxamers F127 and P105 (those with the longest PPO blocks) exhibited more intense excimer emissions signifying a stronger spatial proximity to other pyrene monomers within the lipid core, coupled with a higher excimer/monomer (*e*/*m*) ratio (Table 1).^15^

The *e*/*m* ratio is a secondary feature of the excimer emission where the height of the excimer peak (∼465 to 470 nm) is divided by the first vibronic band, *I*_1_. Previous experiments have showed that *e*/*m* ratios are correlated to the extent of spatial proximity and flexibility of pyrene molecules.^36^ A larger *e*/*m* ratio and excimer emission is due to increased intermolecular coupling of excited pyrene molecules that are more spatially proximal.^36^ On the contrary, Pluronic F68 and L64 SLNs both exhibit lower intensity excimer emissions and *e*/*m* ratios. As previously outlined, our findings suggest adsorption of the PPO block onto the lipid core is sterically restricted at the surface by the PEO chains, however the PEO chains themselves having negligible impact on the internal microenvironment polarity. Therefore, the higher excimer emission and *e*/*m* ratios for F127 and P105 suggests that poloxamers with longer PPO blocks are able to penetrate into the lipid core by a loop formation as highlighted in Fig. 3A. The *I*_1_/*I*_3_ polarity differences indicate that pyrene is more densely packed in SLNs with lower polarity lipid core microenvironments and the *e*/*m* ratio differences suggests spatial entrapment of guest molecules is largely influenced through the poloxamer PPO block length. These findings are of significant importance as this shows that the choice of poloxamer stabilisers used can profoundly impact the internal polarity of SLNs, without causing a significant difference in the size and PDI of the dispersions. Interestingly, this differs to previous studies that correlate an increase in *I*_1_/*I*_3_ with an increase in particle size.^43,47^ It is well studied that pyrene molecules exhibit lower *I*_1_/*I*_3_ values in low polarity organic solvents.^43^ A similar trend is identified within this study as using poloxamers of a longer hydrophobic PPO block length also decreases the *I*_1_/*I*_3_ value. Similarly to organic solvent *I*_1_/*I*_3_ values stated by Flynn *et al.,* our pyrene loaded SLN systems relate to the difference between EtOH (1.36) and THF (1.46) for the longest PPO block length poloxamer (F127; PPO = 65; *I*_1_/*I*_3_ = 1.30) and the shortest PPO block length poloxamer (F68; PPO = 29; *I*_1_/*I*_3_ = 1.40) respectively.^48^ This therefore introduces a new concept of lipid core polarity tuning which has potential to aid drug loading in future formulations.

Blends of poloxamers are commonly used in formulating SLNs and therefore the effect of blending poloxamers of different PPO block length on the internal core microenvironment was investigated (see ESI Table S6† for experimental details). No notable differences in the size or PDI of the resulting SLNs for the blends were observed (ESI Fig. S7†).

When F127 and P105 were blended, it was evident that there was a negligible difference in the polarity environment for the SLNs (ESI, Fig. S8†) which can be accredited to similar numbers of PPO units being able to penetrate the SLN core (PPO units = 65 and 56 respectively). When F68, the poloxamer with shortest PPO block (29), was blended with either of the poloxamer with longer PPO blocks (F127 (65) or P105 (56)), substantial changes in the polarity of the lipid cores was found (Fig. 3). With an increase in the amount of F68 in the blend (PPO = 29) in both cases caused a notable increase in the polarity of the internal lipid core. Fig. 4 highlights that blending F127 with F68 displays a range of *I*_1_/*I*_3_ values from 1.26 at 75 wt% F127 to 1.39 at 0 wt% F127. With reference to Flynn *et al.*, the difference in the experimental values now correlate to *I*_1_/*I*_3_ difference between organic solvent values for IPA (1.21) and THF (1.46) emphasising a controlled variation in the core polarity of SLNs.^48^ This demonstrates tuneability on Compritol 888 ATO cores caused by the stabilisers and should be taken into consideration during formulation development of novel drug loaded SLN systems.

**Fig. 4:**
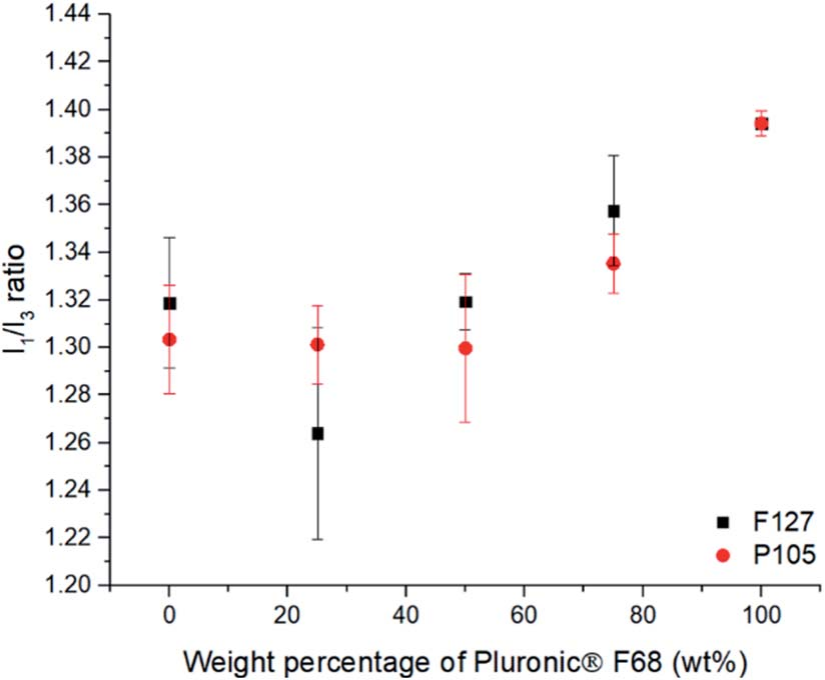
The effect of blending of the different Pluronic® stabilisers on the *I*_1_/*I*_3_ ratio for pyrene loaded SLNs. This shows the effect of varying of the composition of F68 blends with either F127 or P105, showing that increasing the F68 content increases the polarity inside the SLN when using over 50 wt%.

In this study we have shown that pyrene can be used as a fluorescent probe to investigate the polarity inside SLNs. We have found that the length of the PPO block in the stabiliser has a significant impact on the polarity inside the lipid core, with this having a larger impact than the HLB of the stabilisers. This understanding of the influence of the length of the PPO block on the polarity within the lipid core will be important in the design of SLNs. This may offer the potential to tune the internal microenvironment of SLNs in order to enhance their drug loading and drug release behaviour.

## Conflicts of interest

There are no conflicts to declare.

## Supplementary Material

NA-002-D0NA00582G-s001
