# Supplementary material for: Using pyrene to probe the effects of poloxamer stabilisers on internal lipid microenvironments in solid lipid nanoparticles
Source: Nanoscale Adv. 2020 Oct 19;2(12):5572–7. doi: 10.1039/d0na00582g (PMC9417865; doi:10.1039/d0na00582g)
Supplement: NA-002-D0NA00582G-s001 [file NA-002-D0NA00582G-s001.pdf]

Electronic Supplementary Material (ESI) for Nanoscale Advances.
This journal is © The Royal Society of Chemistry 2020

Supporting information for:

Using Pyrene to Probe the Effects of Poloxamer Stabilisers on Internal Lipid Microenvironments in Solid Lipid Nanoparticles

Jessica M. Taylor, Kyle Scale, Sarah Arrowsmith, Andy Sharp, Sean Flynn, Steve Rannard and T.O. McDonald.

Section E1: materials and Methods

Materials:

Pyrene (98% purity), acetone (99.9% purity), 1-propanol (>99% purity), Pluronic F68 (M_w 8,400), P105 (M_w 6,500), L64 (M_w 2,900) and F127 (M_w 12,600) were all purchased from Sigma Aldrich and used as received without further purification. Compritol 888 ATO was kindly gifted from Gattefossé and used as received.

Methods:

SLN synthesis:

The nanoprecipitation method was used to prepare all solid lipid nanoparticle dispersions. For blank SLNs, Compritol 888 ATO (18 mg) was added to 1-propanol (4 ml) before heating to 82°C for 3 minutes until the mixture is homogeneous. The aqueous phase consisted of the chosen stabiliser (16 mg, 0.8 mg/ml) in deionised water (20 ml) that was warmed to 26°C. The hydrophobic phase was aspirated through an 18 gauge hypodermic needle (1.25 mm exit diameter) and injected into the vortex of the aqueous phase through a 21 gauge hypodermic needle (0.9 mm exit diameter). The resultant dispersion was left to stir at 350 rpm on a hotplate stirrer for 5 minutes before analysis.

In order to incorporate pyrene into the samples, a stock solution of pyrene in acetone at 0.1 mg/ml was made. This pyrene solution (340 µL, 0.17 mM) was added to a vial and the acetone was left to evaporate overnight. 1-Propanol (4ml) and Compritol 888 ATO (18 mg) was added to the vial and rolled overnight to ensure total dissolution of the pyrene. The nanoprecipitation procedure as outlined above was carried out.

Solvent controls:

340 µL of the pyrene stock solution (0.1 mg/ml in acetone) was added to 3 separate vials and the acetone was left to evaporate. The different solvent systems (water, 1-propanol and water:1-propanol (5:1)) was added to each of the vials (4 ml/ vial) and left to roll for 48 hours to ensure total solubilisation. The samples were then analysed through fluorescence spectroscopy.

Characterisation:

Dynamic light scattering (DLS) was used to assess particle size and polydispersity of all SLN samples using an Anton Paar Litesizer™ 500 using standard conditions at 25°C. The Litesizer provides polydispersity index values in the range of 0-100% rather than 0-1 as provided on other brands of DLS instrument. Hitachi Spectrophotometer 2700 was used for fluorescence spectroscopy. A wavelength measurement scan was completed from 350-500 nm, excitation wavelength = 336 nm, excitation and emission slit= 2.5 nm and a scan speed of 60 nm/s. All fluorescence measurements for SLN samples were in a mixed solvent system of water: 1-propanol (5:1).

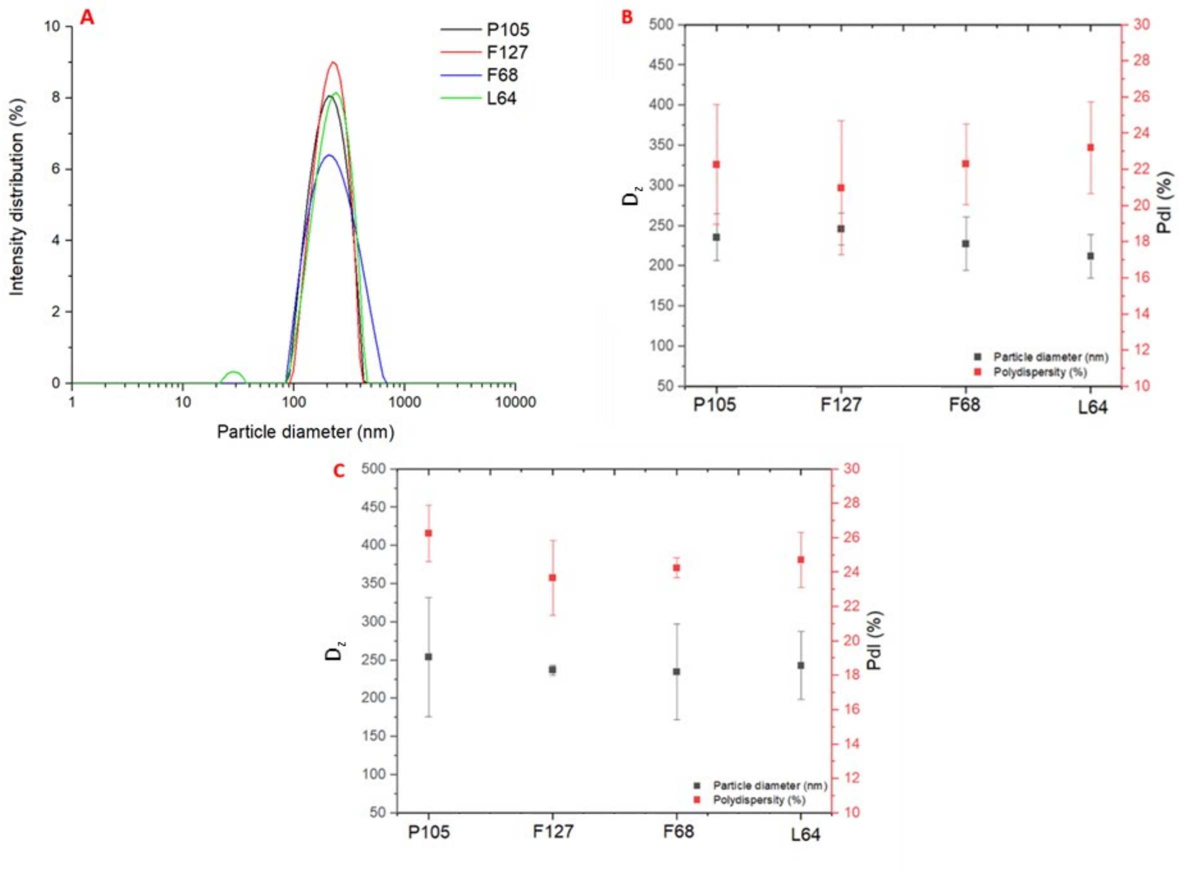

ESI, Figure S1: (A) The synthesised set of blank SLN dispersions gave typically monomodal distribution for the samples. (B) The corresponding numerical data from the DLS for samples (completed in triplicate) shows the D_z (black) and polydispersity of the samples (red). (C) The D_z and polydispersity of pyrene loaded SLNs are shown here. All samples, blank-SLNs or pyrene-SLNs have no significant difference in D_z or polydispersity regardless of the stabiliser used.

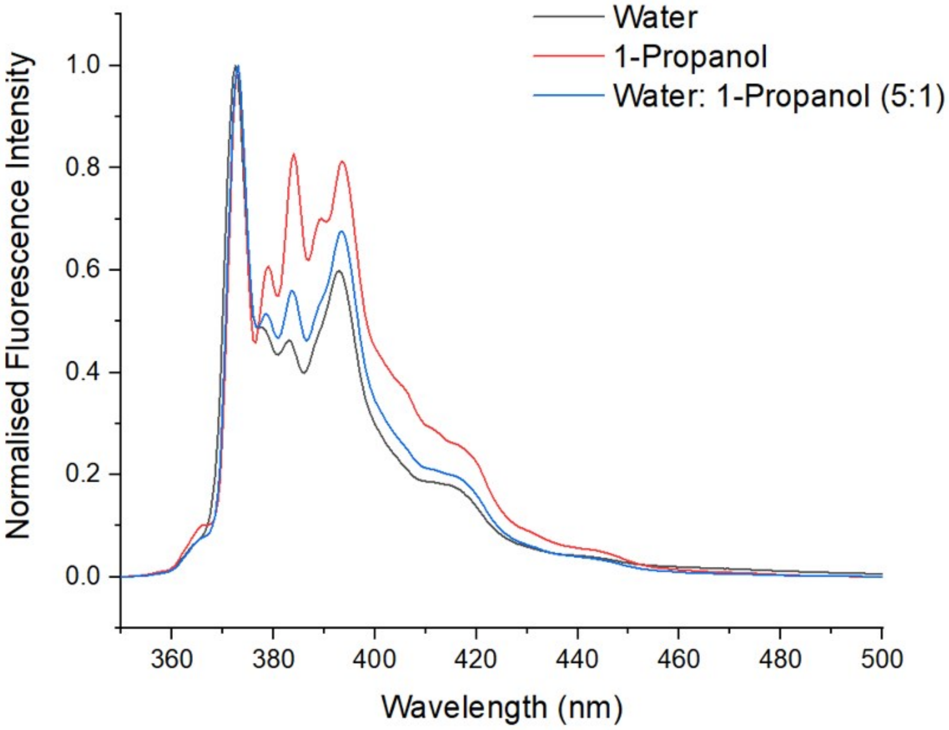

ESI, Figure S2: The fluorescence emission spectra for free pyrene in each different solvent system. The I_1/I_3 values were 2.18 ± 0.09, 1.21 ± 0.002 and 1.78 ± 0.003 and for pure water, pure propanol and the water and 1-propanol mixed (5:1) system, respectively.

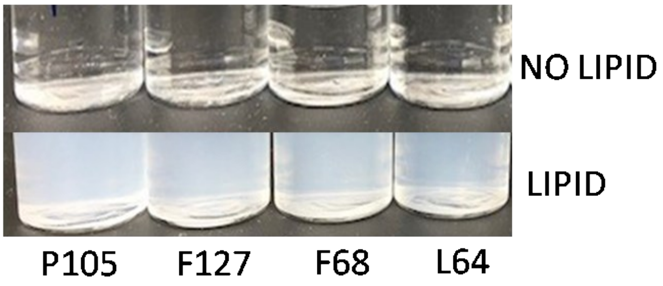

ESI, Figure S3: The aqueous micellar solutions (top) were significantly less turbid than the SLN dispersions (bottom).

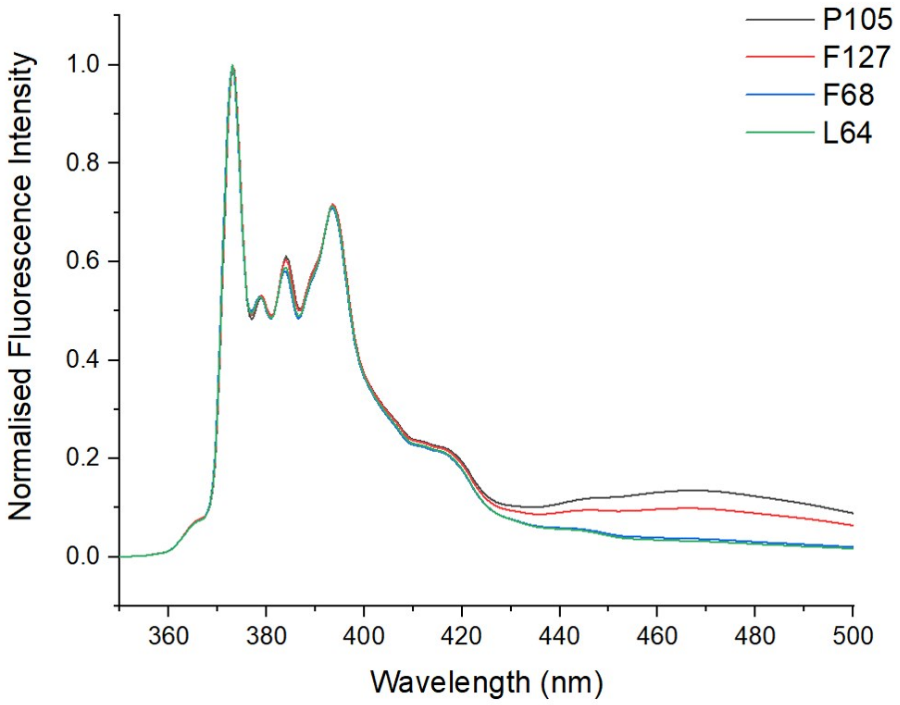

ESI, Figure S4: The fluorescence emission spectrum for aqueous micellar solutions, indicating that some or all of the pyrene molecules had partitioned into the hydrophobic PPO core of the poloxamer micelles.

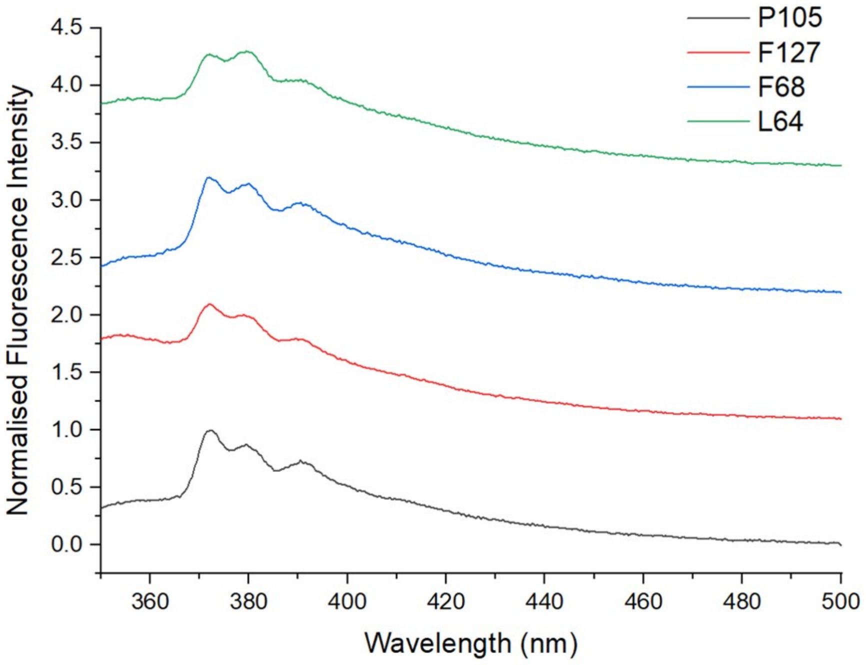

ESI, Figure S5: Fluorescence emission spectra of centrifuged SLN dispersions. The samples were centrifuged through a molecular weight cut off filter (MWCO) of 10,000 kDa. The resulting supernatant became clear from turbid, hence the absence of SLNs. Centrifugation conditions: 9,000 rpm for 60 minutes. The supernatant was analysed for pyrene fluorescence; all samples showed limited pyrene fluorescence suggesting the pyrene molecules resided in the internal lipid core.

ESI, Table S6: Each of the poloxamers were blended in order to identify polarity tuning within the lipid core microenvironment. The commonly used poloxamer, Pluronic® F68 (PPO= 29), was blended with longer PPO block Pluronic's® P105 (PPO= 56) and F127 (PPO= 65).

	**P105 (wt %)**	**F127 (wt %)**	**F68 (wt %)**
**P105 (wt %)**	100	50:50<br>25:75<br>75:25	50:50<br>25:75<br>75:25
**F127 (wt %)**	-	100	50:50<br>25:75<br>75:25
**F68 (wt %)**	**-**	**-**	100

The table shows the weight percent ratio of mixed poloxamers used to form the SLNs. All SLNs were formed with a total concentration of poloxamer in the aqueous phase at 0.8 mg/ml, 0.8 w/v%.

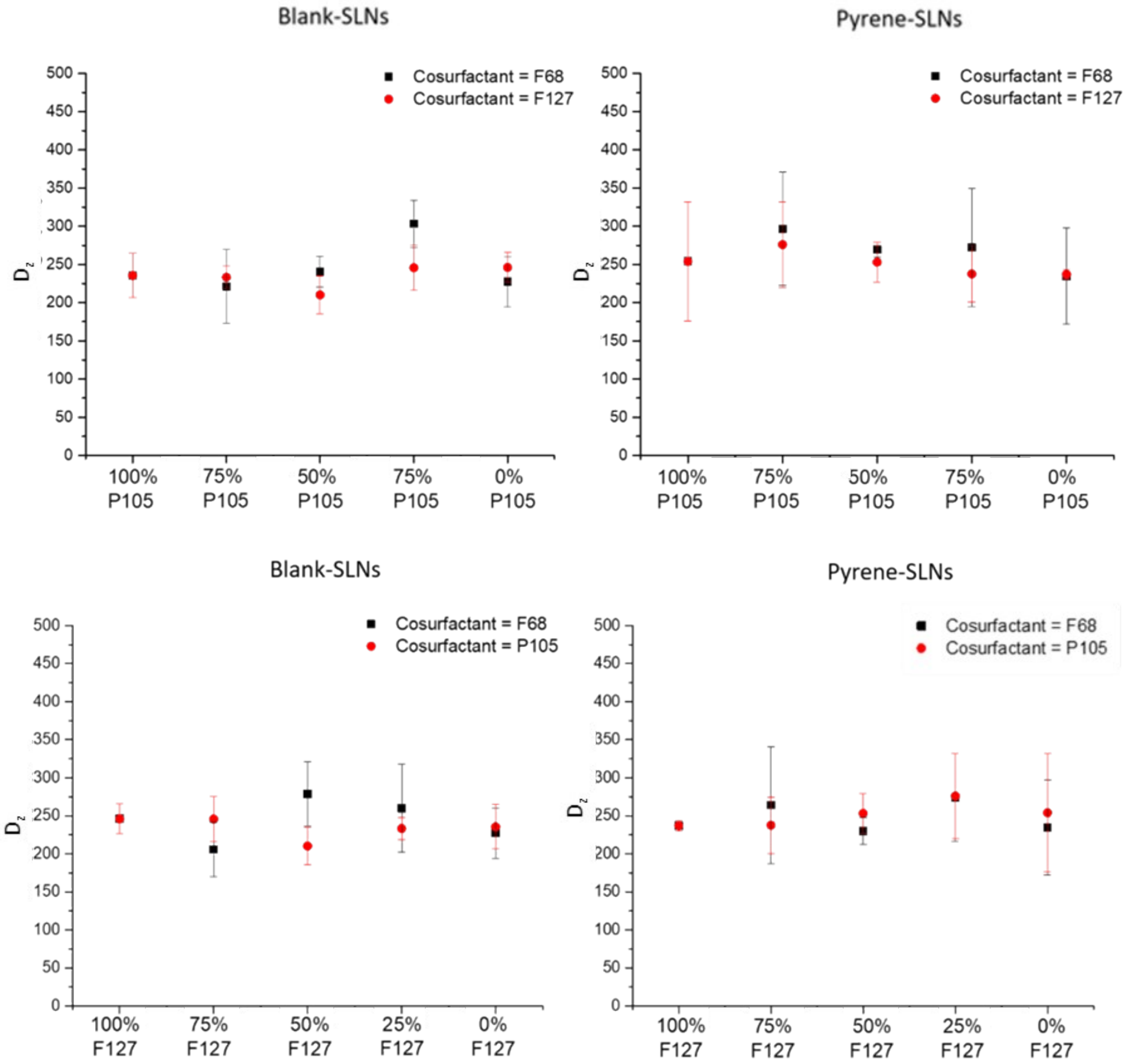

ESI, Figure S7: The D_z and polydispersity data for SLNs stabilised by blended poloxamer stabilisers. The left-hand column represents lipid only SLNs denoted as “blank SLNs”, and the right-hand column represents pyrene loaded SLNs. The four graphs above show all potential blends and ratios of poloxamers that were investigated (P105:F127, P105:F68, F127:F68). There was negligible difference in the D_z or polydispersity throughout these samples.

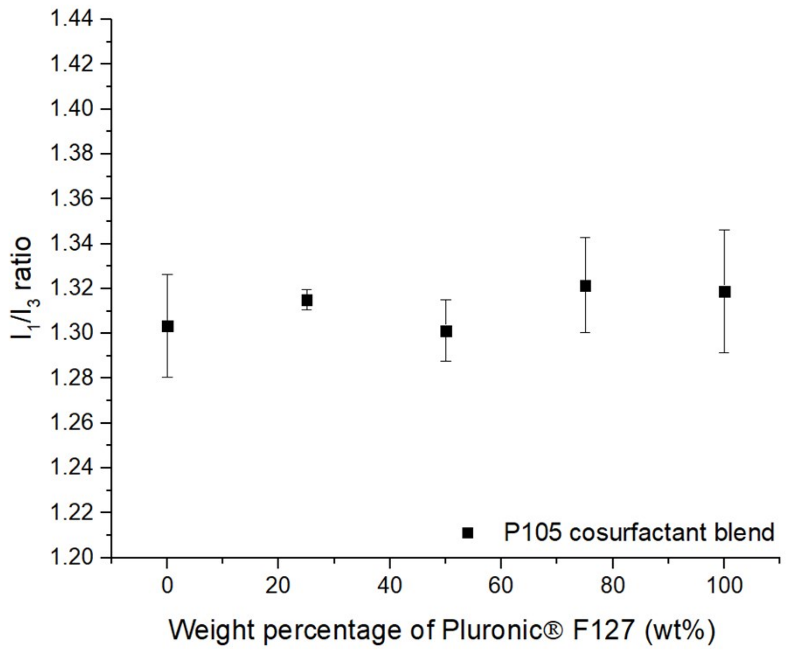

ESI, Figure S8: The effect on the internal lipid core polarity when blending Pluronic® F127 and P105 to form mixed poloxamer stabilised SLNs. As shown in here, there is not notable difference between the I_1/I_3 values, hence no significant difference in the internal core polarity of these SLN systems.
